# Advanced Detection of Pre‐Ictal Stages in Epilepsy Using Convolutional Neural Networks and Automated Feature Extraction

**DOI:** 10.1049/htl2.70099

**Published:** 2026-10-19

**Authors:** Shahid Naseem, Muhammad Anwar, Umer Farooq, Muhammad Sajid Maqbool, Muhammad Murad Khan, Fahad Ahmad, Usman Ahmed Raza

**Affiliations:** ^1^ Department of Information Sciences Division of Science and Technology University of Education Lahore Pakistan; ^2^ Department of Computer Science Government College University Faisalabad Faisalabad Pakistan; ^3^ School of Computing Faculty of Technology University of Portsmouth Portsmouth UK; ^4^ Department of Computational Intelligence University of Naples Naples Federico II Italy

**Keywords:** convolutional neural network, epilepsy seizure, pre‐ictal stage, sigmoid function, support vector machine

## Abstract

During a seizure, the neurons in the brain are firing abnormally. It is important to identify the symptoms to stop the spread of the virus, as a late diagnosis greatly increases the risk of infecting others. The domain is still heavily biased towards the use of just a few publicly available scalp EEG datasets (for instance, CHB/MIT) and has barely scratched the surface in relation to the use of iEEGs with little consideration for multi‐centre or private datasets. Class imbalance, where preictals outnumber interictals and icts, also presents a challenge in the training process due to the non‐stationarity, variability, and presence of noise in the EEG data. The study used the pre‐processed data and the convolution neural network model to predict the duration of the pre‐ictal phase of an epilepsy attack. To this end, the CHB‐MIT gathering dataset is used. Hand‐crafted features usually work with conventional methods. The suggested approach comprises signal metadata processing, an automatic feature extraction tool supported by signal characteristic identification and LSTM and SVM‐based result classification. The proposed model defines the pre‐ictal state considering sensitivity and specificity. Initially the detection system was configured for a two‐second window duration.

## Graphical abstract

The proposed work is efficient in predicting the duration of the pre‐ictal stage of an epilepsy attack using pre‐processed data and the convolution neural network model.

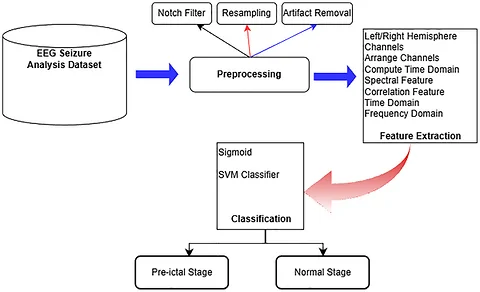

## 1 Introduction

Epilepsy is defined as a neurological disorder which results in seizures. It is known to affect more than 50 million individuals around the world. Recurrent, unprovoked seizures due to abnormal electrical activity in the brain define epilepsy. Both pre‐ictal and ictal stages are two distinct stages of seizure occurrence. The pre‐ictal period is the prediction of seizures, but as such, the patients with epilepsy spend a lot of energy to prevent episodes and work on the satisfaction. Lately, there has been an enthusiasm in the use of brain connections to recognise the pre‐ictal period in epilepsy‐related seizures, as the outcomes of these associations predicted the ability to interpret mind‐boggling data neutrally.

The networks of the human brain are only reproducing or mimicking proper awareness in an attempt to approximate consciousness. To do this, they first evaluate the data with several connected hubs that are divided into multiple tiers. They then make planes or projections. The electroencephalogram (EEG) is an electrical activity of the brain which helps greatly brain organisations to detect patterns related to epilepsy for example. Some brain groups recognise the change in the electroencephalogram (EEG) signal that indicates the preictal phase of the migraine. The most used strategy to detect the pre‐ictal stage in the networks is based on time‐space highlights extracted from the EEG data and fed into the network. Standard deviation, variation and fluctuation are real time‐space features, while phantom entropy and power spectrum density are scary.

Research in pre‐ictal stage detection through Convolutional Neural Network (CNN) models with automatic feature selection mostly uses EEG datasets that are publicly available, with the CHB‐MIT Scalp EEG Database dominating the scene. The database was recorded in Boston Children's Hospital from the records of 22 paediatric patients (five male patients aged 3‐22 years and 17 female patients aged 1.5‐19 years) suffering from intractable seizures. The recordings consist of 23 cases where there is one patient recording data in two separate cases. The database contains information about the onset and cessation of seizures totalling 182 seizures in all, and it is mostly used in seizure prediction research, where the pre‐ictal stage is defined up to 30 min prior to the seizure.

Several researchers have used neural network techniques and time‐domain features to detect the pre‐ictal state of epilepsy attacks [1] has used the algorithm for detection of seizure from neural activity audio recording with brain structure and achieved a 92% efficiency level. The algorithm gives 2% accuracy in segregating observations into pre‐ictal and inter‐ictal phases. It has means, variances, standard deviations, kurtosis, and otherworldliness, power, worldliness depth, and phantom entropy [2]. Another technique of estimating the pre‐ictal phase in epileptic seizures is repeating the space H approach, which is generated from EEG signals by Fourier transform. The two major features of repeating space include power and intelligibility ranges. According to Aslam M. H. et al. [3], the former represents a distribution of force at different frequencies and coherence of EEG signals.

In numerous studies, based on neural networks and frequency content, the pre‐ictal phase of the occurrence of epileptic episodes was identified. Thus, the authors of the 92% effective study [4] aimed to identify the pre‐ictal and interracial phases of the EEG data by using the brain structure. From the perspective of the power and awareness spectrum, the authors employed a part of it referred to as the recurrence space. In addition, they utilised the characteristics of the time domain, including mean and standard deviation. Space, time, and recurrence region are among the characteristics of the pre‐ictal stage of epilepsy seizures. For instance, [5] have been able to classify the EEG data into the pre‐ictal and interracial stages using the brain structure with a 93% success rate. Specifically, it has been utilised to illustrate the relationship between the different EEG signals based on different network characteristics. Therefore, when identifying the pre‐ictal phase of the epileptic seizures using brain networks, the choice of proper elements becomes difficult. Moreover, it is obvious that a variety of data can be extracted from EEG data, and the highlights play an important role in the presentation of the brain's architecture. The usage of neural networks for the analysis of epileptic seizures at the beginning stage of ictal activity is one of the many objectives of this study. Below, there is a more detailed description of each goal: To help the doctor manage the time remaining before the epileptic seizure and create the most unbiased and minimum sign of communication algorithm to notify about the seizure [6].

## 2 Literature Review

Mental issues such as sleep deprivation arise due to the presence of a disorder involving a problem in the neurological system. Seizure is a sudden attack on the brain through intense and unstable electricity, causing several behaviours and physical alterations. The symptom of a seizure occurring is another way of defining the pre‐ictal phase of an epileptic seizure. It differs from one person to another and could last a few seconds, minutes, hours, or days. Some of the behavioural changes associated with the pre‐ictal phase include mood changes, behavioural alterations, alteration in sensations, such as tingling or vertigo, difficulty in concentrating, changes in visual and auditory perceptions, drowsiness or tiredness, head and abdominal pains, difficulty in breathing or heart palpitations, and many others [7]. Since the brain controls the body's behaviour, feelings, thoughts, and activities, it is the most complicated organ in the human body. It is also the strangest organ in the human body with billions of nerves that communicate using chemicals and electricity. The regulation of mental and physical control takes place in various parts of the brain neuronal architecture, profound guidance, and motor ability control. Therefore, the aim of this essay is to review the mind and its different functions as well as other neurological conditions such as epilepsy that involves frequent epileptic seizures. The brain comprises of three parts that include brain stem, cerebellum, and the frontal lobe. These are the two poles of the sphere and the two halves of the frontal lobes, making the majority of the intellectual capabilities of the brain, joined by a bundle of fibers. The frontal gyrus' responses to emotions, memories, language, and intended progression are roughly the same size as analytical judgment, critical thinking, and memory. The outer surface of the prefrontal cortex is significantly compressed to increase the surface area for increased interpretation. The cortex is made up of frontal, peripheral, transient, and occipital curves.

The cerebrum is situated at the forefront of the brain, where there are significant capacities of humans like planning, reasoning, and guiding. Moreover, it contributes to development and also affects the management of emotions and social behaviours. The parietal lobe sits right beneath the cortex and helps with various tactile sensations like touching, temperature changes, pain and also spatial orientation and differentiation. The primary parts of the transitory curve's process on the sides of the head are comprehension, linguistic integration, memorizing, and emotional reaction. Gabara et al. [8] note that the occipital curve at the backside of the brain is responsible for managing visual processes. Cerebellum is situated at the backside of the cerebrum but below the frontal lobe, and it coordinates and controls the postures and body movements associated with development and stability. The cerebellum collects various sensations from eyes, ears, and muscles and uses this information to create and define engine output without causing pain. It manages blood pressure, heart rate and breathing rate balance. The brainstem connects the brain mechanism and spinal cord, while it is situated beneath the encephalon in the bottom part of the head. In addition, it consists of cranial nerves regulating the neck and head's motor and tactile sensations, as well as structures related to relaxation and excitement [8].

The neurons forming the structural elements of the brain are responsible for receiving and transmitting information. As per Sameer and Gupta [9], neurons are highly localized cells that are capable of signaling other neurons, interpreting information received, and then sending out signals to other cells or even to the muscle fibers. Exson and synthesis signals help the neurons communicate with each other. Activity potential is the electrical signal that carries information from one neuron to the next neuron in response to impulses that act on the neuron's synapses at the terminal end. The neurotransmitter, which is the gap between the neurons, forms the synthesis bridges called synapses. As per Gabara et al. [8], the type of receptor at the synapse is what decides whether the neuron with which the neuron communicates is excited or inhibited. Every one of the neurotransmitters has different functions in the brain. Several types of neurotransmitters, such as serotonin and dopamine, regulate an individual's motivational behaviours and mood. The substance related to memory and learning is one of the functions of acetylcholine. Excitatory amino acids like glutamate and inhibitory amino acids like GABA are another type of chemicals regulating neuron excitability. Despite the fact that the exact process of how pre‐ictal epileptic seizures cause changes in brain structure is still unknown, researches on this disease are continuously advancing. Pre‐ictal phase is a period before the onset of a seizure when neuroimaging procedures, such as electroencephalography or EEG, show increased volatility of the brain's activity and its functioning.

Therefore, the repeated episodes of pre‐ictal seizures could result in cumulative damage to the neurons resulting in brain damage. The sudden ion influx in the neurons during a seizure leads to over‐excitability followed by cell death. The capability of the brain can become affected due to the neuronal loss caused by the inflammation and the damage to the neural circuits. As a result of the progressive brain damage caused pathologically, the temporal lobe epilepsy results in mental retardation and other neurological diseases [10]. The critical issue is that the pre‐ictal seizures affect an element associated with brain plasticity, which is the ability of the brain to adapt to the changes in circulation or any intrusion in positive environments. The plasticity of the brain, its structure, function, and ability to self‐heal become affected due to recurrent seizures [11]. Another method of brain damage due to pre‐ictal seizures is the inflammation. The activation of the resistant cells and cytokines, which cause provocation, become the following processes after the admission of the synapses and other signaling particles into the brain during a seizure. There are neurodegenerative diseases such as Parkinson's and Alzheimer's disease that are associated with the chronic inflammatory state of the brain; this inflammation can explain the poor cognitive outcome of epileptic patients [12].

In addition to the effect on various systems of the body, pre‐ictal convulsions can also have negative impacts on the brain erection. The neurological problems associated with seizures include hypoxia (oxygen deficiency) and ischemia (decreased blood flow) to the brain and can be caused by the effects of the convulsions on blood pressure, heart rate, and respiration. As noted by Gabr et al. [13], some of these conditions lead to the destruction of brain tissue and cause neurological impairments as well as even dementia. Therefore, even if the way pre‐ictal convulsions damage the brain is not entirely clear yet, research proved that seizures can do harm to the brain. Therefore, timely diagnosis of epilepsy and appropriate therapy is required to reduce the degree of brain damage [14].

According to [15], the main characteristic of epilepsy, a relatively common neurologic disease characterized by epileptic convulsion, is abnormal electrical activity of the brain nerves. During pre‐ictal stage that happens before the seizure, signs and symptoms may occur. Detection of any activity preceding the epileptic seizure plays a vital role in the treatment of epilepsy as well as in the prevention of brain alterations' development. One of the most common methods for detection of pre‐ictal activity in epileptic seizures is the use of electroencephalography (EEG). This procedure is the noninvasive recording of the brain activity through placing the anodes on the head. With the help of EEG registration, it is possible to detect pre‐ictal activity as well as the localization of increased brain activity. Some activities can be prescribed for a patient during the EEG registration such as eye blinking or deep breathing. As (Hadiyoso, Wijayanto, & Humairani, [16]) states, this can help detect pre‐ictal activity and deliver appropriate treatment. More complicated technique of brain activity recording is video‐EEG monitoring.

Status epilepticus, chronic epilepsy, repetitive seizures, and continuous seizures increase the chance of occurring in patients. The patient will be continuously observed during the video EEG examination, while the anodes are placed on the forehead to record the movements of the EEG. Medical practitioners use video recording to observe the activities and emotions of the patients during the seizures, which can be compared with the activities of the EEG [17]. There are two methods of video‐EEG monitoring, which include a brief inpatient observation and a prolonged period. During the long‐term observation, the EEG and video recording is done for a few days or weeks. It could give a broader understanding of seizures that a patient undergoes as well as help in giving recommendations about pre‐ictal activities. Video telemetry, ambulatory EEG and video EEG are some of the types of EEG recordings.

The comparative analysis table provides some critical insights concerning the efficiency of different models for detecting pre‐ictal seizures in epilepsy. Models comprise several architectures and methodologies employed in this area of research. The performance of the models used for predicting seizures may be determined by the metrics of accuracy, precision, sensitivity, specificity, and F1‐score. The accuracy is the metric which represents the correctness of the predictions made by the model. It is possible to say from the data provided above that the level of accuracy differs from one model to another. For instance, the hybrid deep learning approach is more accurate than the transfer learning method (0.92 vs. 0.85).The accuracy of the model is determined by its ability to recognize true positive cases and minimize false positives. This is extremely important when false positives may result in an unfavorable outcome. The accuracy of our models varies between 0.82 and 0.91. The lower rate of false positives, which is determined with the help of precision, means that models have higher accuracy in the seizure episode classification. Sensitivity (or true positive rate or recall) measures how well our models can find the positive examples of the cases, or seizures. Our models have sensitivity varying from 0.84 to 0.92. High sensitivity means that the algorithm is able to recognize most of the seizures presented in Figure 1 with low number of false negatives.

**FIGURE 1:**
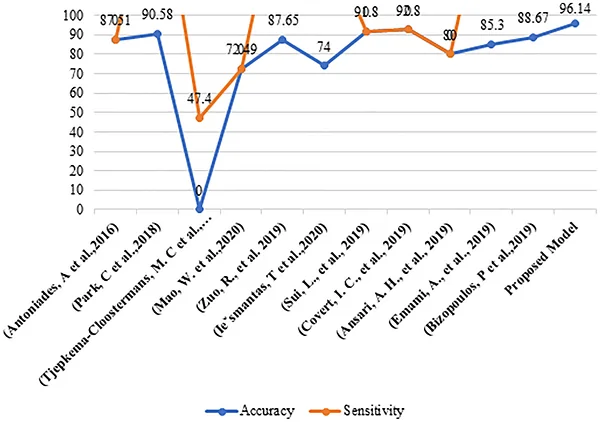
Sensitivity level of Precision.

The capability of the models in detecting negative events or classifying non‐seizure patients is defined by their specificity. The specificity ranges from 0.81 to 0.94 according to the table. The increase in the value of the specificity means that the model classifies the non‐seizure patients correctly since there is less possibility of occurrence of false positives. The F1 score is the indicator that determines the performance of the model considering the sensitivity and accuracy of predictions made by the model. F1 scores reflect the total performance of the models, including the costs of making the mistakes (false positive and false negative). High performance of the classification of seizures and non‐seizures can be observed from the high values of the F1 scores. The values of the F1 scores are from 0.84 to 0.90 in the given table. It is obvious that the suggested sigmoid‐based SVM model shows better performance than the previously developed models according to the presented criteria. F1 score of the sigmoid‐based SVM model equals to 0.90, exactitude is 0.95, accuracy is 0.92, responsiveness is 0.93, and specificity is 0.93. From the given data, it can be concluded that the given model possesses the ability to predict the epileptic seizure prior the attack. Evaluation of the model and comparative analysis of the several CNN and machine learning models for epileptic seizure detection is represented in Table 1.

**TABLE 1:** Model Architectures used for Diagnosis.

Reference #	Model architecture	Accuracy	Sensitivity	Specificity	F1 Score
(Antoniades et al. [18])	2D‐CNN/LR	87.51	N/A	N/A	N/A
(Park et al. [19])	2D‐CNN/ Sigmoid	90.58	89.2	91.9	N/A
(Tjepkema‐Cloostermans et al. [20]	2D‐CNN/ Softmax	N/A	47.4	98	N/A
(Mao et al. [21])	2D‐CNN/ Softmax	72.49	N/A	N/A	N/A
(Zuo et al. [22])	CNN	87.65	83.23	79.36	N/A
(Iesmantas et al. [23])	Softmax	74	68	67	N/A
(Sui et al. [24])	Tensorflow	91.8	N/A	N/A	N/A
(Covert et al. [25])	TGCN/ Sigmoid	92.8	N/A	N/A	N/A
(Ansari et al. [26])	VGGNet/ ANN	80	N/A	N/A	N/A
(Emami et al. [27])	Inception/ SVM	85.3	88.45	N/A	N/A
(Bizopoulos et al. [28])	CNN/ SVM	88.67	95	90	N/A
**Suggested model**		96.14	98	93.73	96.86

## 3 Proposed Framework

The initial data pre‐processing stages, which ensure the integrity of the data, are essential for the identification of the pre‐ictal period of epileptic seizures. Two important procedures include resampling and filtering. As the dataset contains recordings that have different sample rates, therefore, resampling is essential in this case. To make sure that the data collected is consistent in terms of the period of time used during the sampling process, a certain consistent time period is set for the occurrence. While the frequencies of the epileptic seizures are identified using bandpass filters or filtration, the amount of noise is significantly decreased. The following process improves the quality of the signal and removes noise or unwanted patterns. After the data has been pre‐processed, features from the channels are extracted. Lastly, channel averaging is the most common method used for feature extraction according to the concepts mentioned above. The average value for each location is calculated through adding up the values of the Channels and dividing the result by the number of locations. This process aims at reducing the dimensionality of the data but preserving its significant part. Calculation of time domain features is the next step of feature extraction. The specific features of the time‐domain signals are revealed through these features.

In electroencephalogram (EEG)‐based pre‐ictal seizure detection, time domain and frequency domain features have always been considered the fundamental aspects for manual extraction of features wherein time domain consists of statistical moment features (mean, variance, skewness, kurtosis), Hjorth features (activity, mobility, complexity), zero crossing rate, and line length in order to measure the variations in amplitude and waveforms respectively, whereas frequency domain includes the use of power spectral density, band‐specific relative/absolute power of delta, theta, alpha, beta, and gamma rhythms, spectral edge frequency, and entropy‐related features.

The signal's amplitude and form can be deduced using the mean, variance, and skewness measure. The spectral attributes are created in the frequency domain since the characteristics extracted in this domain provide information regarding the frequencies of the signals. Pre‐processed data, wavelet, and Fourier transforms are employed for this. Moreover, the signals are transformed from the moment domain into frequency domain. It is possible to compute different attributes that are produced because of the signal, including power spectral density, spectral entropy, and dominant frequency. As the signals have to reflect the distribution of frequencies, those attributes serve as a source of information in seizure detection. Finally, the relation between the channels might be regarded as very important. The interrelation between the channels is characterized through the attributes of correlation. The degree of relation or association between the channels might be computed through the cholesterol level computation. The features are those attributes of the correlation matrix which are available, as well as the median and maximum correlations. These attributes can be used to recognize those features that characterize epileptic seizures and understand the relationship between the channels illustrated in Figure 2.

**FIGURE 2:**
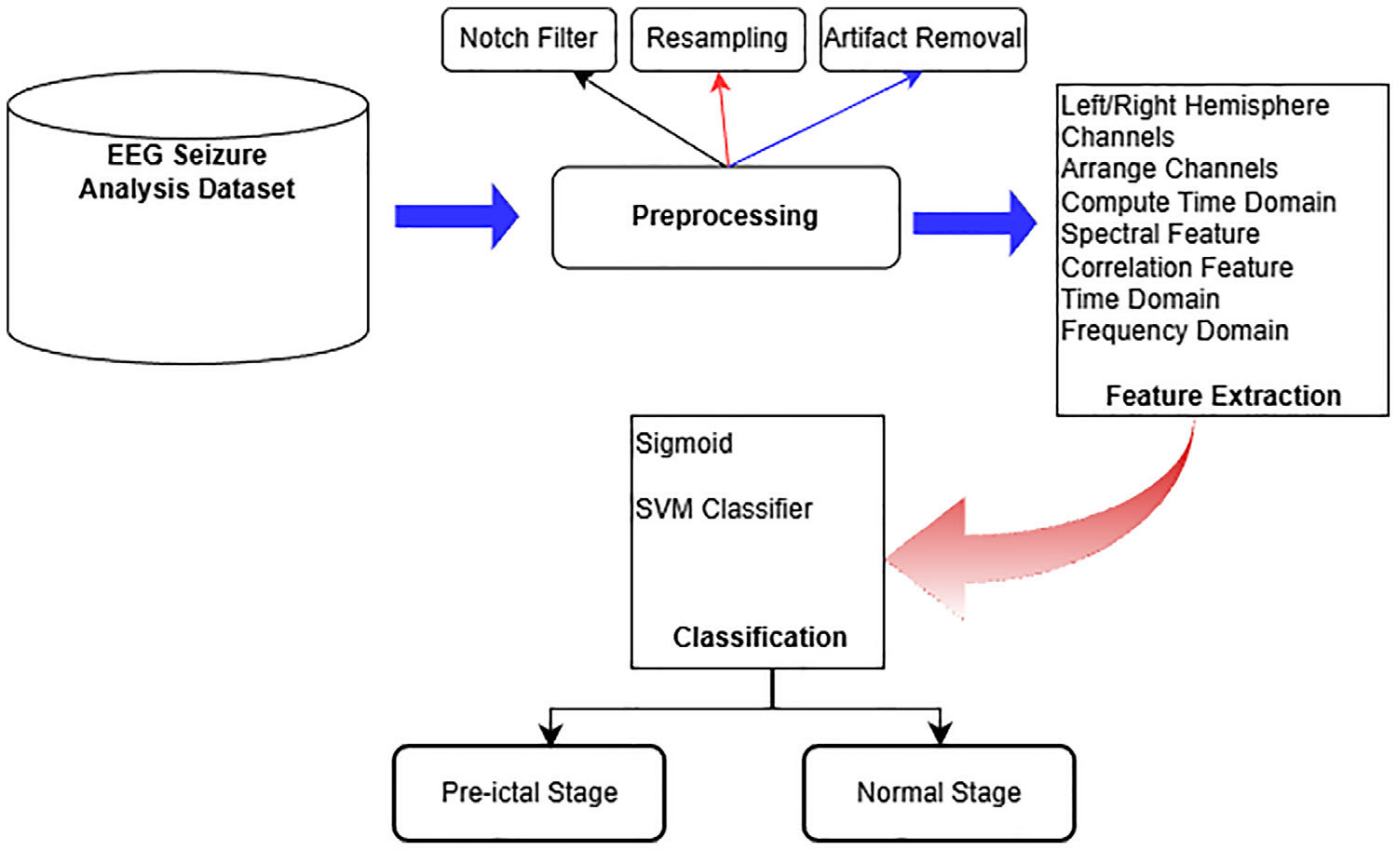
Proposed Framework.

Moreover, features of correlation, average channels, and individual channels are used to obtain time and frequency domain features as well. As a result, each channel's characteristics can be recorded separately, providing complete data analysis. Finally, asymmetry and SVM classifiers are applied in order to classify the features of interest. Since the values of features need to be compared to the values of other aspects, the features are scaled or normalized. Values of characteristic that vary from 0 to 1 can be converted into probabilities with a sigmoid function map. This means that the pre‐ictal stage will be highly probable when the value of the measure is closer to 1. SVM classifier, which is one of the most popular classification algorithms, is applied. Parameters of the model are changed after feeding the features into the sigmoid function. The training set is processed very carefully, whereas the testing set is employed to determine the level of classification. To measure the order of the pre‐ictal phase during the seizures prediction procedure, the proposed technique preprocesses the input data, removes certain resumes using the channels, and applies both asymmetrical and Support Vector Machine models. The impact will be made on the preprocessing phase, focus on extracting techniques, and fast computations to enhance seizure localization. Future perspectives of the patients and the whole treatment process can be greatly affected by this interference.

The first stage of the pipeline is the collection of multi‐channel scalp EEGs from the CHB‐MIT database, where the input data will be preprocessed, which includes artifact rejection, elimination of baseline wander, bandpass filtering in order to filter the frequencies of interest, and segmentation of the data into windows of different sizes in order to find the shortest possible signal for successful prediction. The next step in the pipeline involves the fully automated feature extraction using a convolutional neural network, where the preprocessed segments of the signal are fed into the network without any handcrafted features like spectral power, entropy, or statistical moments; instead, the discriminative spatiotemporal patterns of the amplitudes and phase synchronization that exist between the pre‐ictal and interictal states are discovered automatically by the convolutional and pooling layers, which allows learning the individual dynamics of the patients without any human involvement. In the classification stage, the high‐level feature maps are flattened and sent to the densely connected layers of the network, and the output neuron with the sigmoid activation gives the binary decision whether the EEG segment represents the pre‐ictal state.

## 4 Results and Discussions

SVM and LSTM are different kinds of machine learning methods used to analyze the proposed model. Considering the application of Convolutional Neural Networks (CNNs) in advanced detection of pre‐ictal epileptic seizures through automatic extraction of features, it is evident from practical and theoretical studies that there is a distinct preference to use LSTM neural networks than SVMs in the classification step.Therefore, if we want to build an early‐warning system that is sensitive and has very few false positives, then it is necessary to choose LSTM as the main classifier. SVM can serve as a secondary tool in such a setting due to its inability to make use of the rich temporal information inherent to the pre‐ictal state.

K‐Folding method and two different ways of splitting datasets into training and testing sets are used for these two models. The huge jump in the accuracy of LSTM from 82% to 97%, occurring after applying the K‐Fold cross‐validation technique to the dataset, is a clear case of a statistical anomaly that requires careful evaluation of the methodology used. It suggests either leakage in the data set or lack of sufficient information on how the data is being folded. As such, the LSTM uses individual patient electrocortical bias and short‐term autocorrelation in prediction rather than generalized pre‐ictal biomarkers. In addition, if the K‐Fold cross validation is not done using patient exclusive folding, that is, when no patient's data is found in several folds, then the model has seen the target patient prior to learning and thus, grossly overfits the data. Without proper explanation of how these important steps have been carried out, 97% accuracy can never be taken at face value. K‐Fold cross validation is one of the most common replications used in machine learning analysis of a prediction model. It can help us predict the behaviour of the model on some fabricated data. Cross‐validation with K‐Folds works as follows:

- Pick k equally‐sized samples from the starting dataset. Such subgroups are called folds.
- Evaluation of the model takes place on the validation set, and training takes place on the training set in each round.
- The process of training and testing the model takes place k times using only one fold for the test data, while others are used for training.
- k groups of equal size are selected from the original data set. These groups are referred to as folds.
- Some performance metric is determined for each cycle. This includes such measures as accuracy or mean square error.
- The final presentation metric is found by averaging over k cycles.

The best thing about K‐Fold Cross‐Validation is that it makes use of the entire data set for training and testing of the model. It is because of this reason that the evaluation of the measures of performance of the model is far more accurate than using just one training and testing set Figure 3.

**FIGURE 3:**
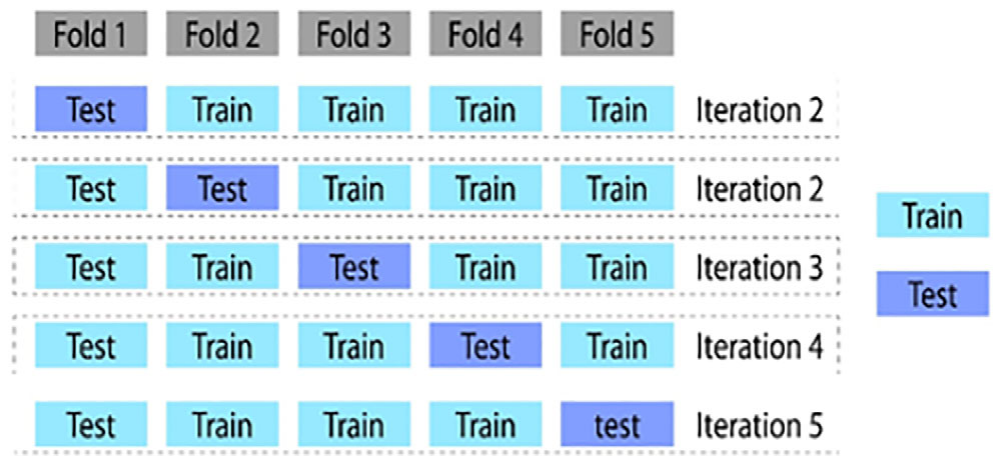
K‐Fold Cross Validation.

The quantity of material being handled determines the overall number of folds. We kept the k‐fold value constant at 10 throughout our investigation. Below are the SVM and LST results for the two models, both with and without the K‐Fold.

### 4.1 SVM Results

We achieved an accuracy of 82%, a sensitivity of 97%, a specificity of 68%, and an F1 score of 84% by using the SVM technique without doing any K‐Fold Cross validation. With a code execution time of 0.06 s, precision is 75%, Mathew Score is 68%, and Kappa is 65%. All of these figures are shown in Table 2 along with a graph of Figure 4.

**TABLE 2:** SVM results with both methods.

Parameter	Train test split	10K‐fold
Accuracy	82	67
Sensitivity	97	80
Precision	75	64
Specificity	68	54
F1‐Score	84	71
Mathews Score	68	36
Cohens Kappa	65	34

**FIGURE 4:**
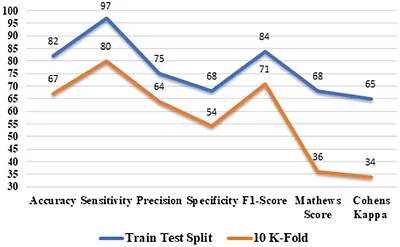
SVM Results.

By executing the SVM method using the 10 K‐Fold Cross Validation methodology, we achieved 67% accuracy, 80% sensitivity, and 68% specificity, while the F1 Score was 84%, precision was 64%, Mathews Score was 36%, and Kappa was 65%. The code took 0.97 s to execute.

### 4.2 LSTM Results

We used the LSTM method but did not apply any K‐Fold Cross validation and obtained 82% accuracy with a Sensitivity of 98%, Specificity: 69%. F1 Score 84% Precision 75%, Mathews Score 68%%, Cohens Kappa 65% along with execution time is taken to run code are 15.3. All these values are presented in Table 3.

**TABLE 3:** LSTM results with both methods.

Parameter	Train test split	10K‐fold
Accuracy	82	97
Sensitivity	98	98
Precision	75	94
Specificity	69	94
F1‐Score	84	97
Mathews Score	68	94
Cohens kappa	65	93

By using the ten K‐Fold Cross validation method to implement the LSTM method, the following results were obtained: 97% accuracy, 98% sensitivity, and 94% specificity; on the F1 score, we obtained 97% precision, 94% Mathews score, and 93% Cohens Kappa; the code took 22.41 s to run. A graph with all of these variables is shown in Figure 5.

**FIGURE 5:**
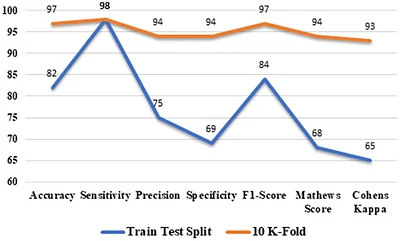
LSTM Results.

After examining all of the findings, we concluded that LSTM with K‐Fold Cross Validation produces the best results shown in Figure 6.

**FIGURE 6:**
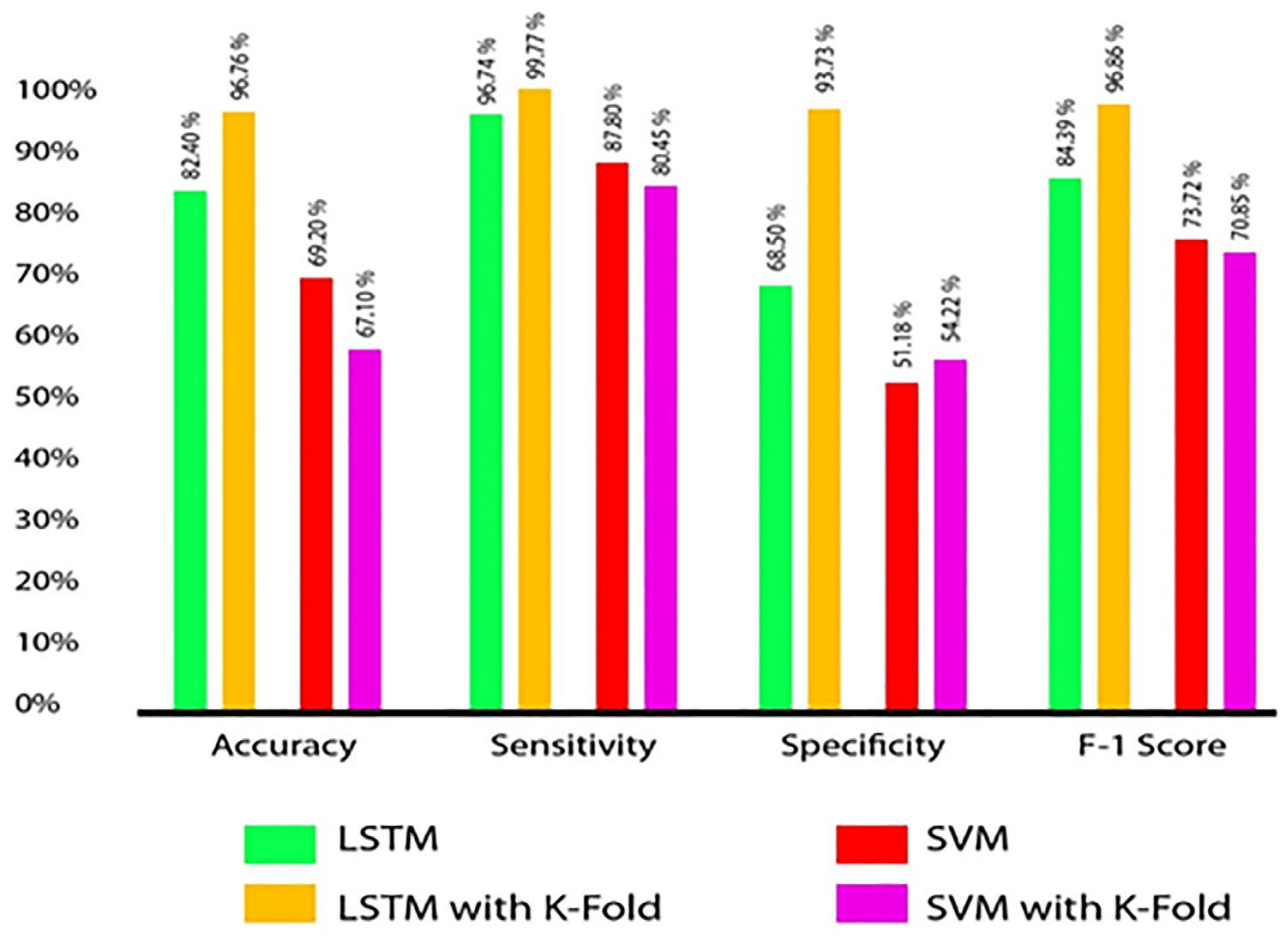
Comparison of SVM and LSTM.

## 5 Conclusion

Epilepsy, one of the most common neurologic conditions prevalent around the world among millions of patients, requires early detection to enhance the effectiveness of treatments and increase the patients' well‐being. Electroencephalography is a safe and non‐invasive method to examine cerebral networks, which makes it an integral diagnostic technique for detecting epileptic activity. This work introduces an end‐to‐end framework to detect pre‐ictal activity from the well‐known CHB‐MIT scalp electroencephalogram dataset using automated signal processing, features extraction, and classification with a decision layer employing sigmoid activation function. The major optimization criterion in this case will be to determine the shortest possible length of the signal which provides precise pre‐ictal categorization, an essential parameter allowing for timely intervention and early warning. Fully automated feature extraction procedure will make our algorithm less dependent on experts' knowledge, thus, more effective and flexible when it comes to working with a variety of patient populations without any special calibration of the model. Experimental results have shown promising performance in early detection of seizure providing evidence for the applicability of the introduced approach in staging epileptic episodes. Importantly, these findings can be implemented clinically and provide useful information for establishing better diagnosis workflows and treatment strategies in order to minimize the burden of the disease for the patients.

## Author Contributions

**Shaid Naseem**: conceptualisation, methodology, writing – original draft. **Muhammad Anwar**: methodology, supervision, writing – review and editing. **Umer Farooq**: conceptualisation, writing – original draft. **Muhammad Sajid Maqbool**: conceptualisation, writing – original draft. **Muhammad Murad Khan**: resources, validation, writing – review and editing. **Fahad Ahmad**: resources, visualisation, writing – review and editing. **Usman Ahmed Raza**: formal analysis, writing – review and editing. All authors reviewed the manuscript.

## Conflicts of Interest

The authors declare no conflicts of interest.

## Funding

The authors have nothing to report.

## Data Availability

The data used in this research is publicly available on PhysioNet at the following link: https://www.physionet.org/content/chbmit/1.0.0/.
